# Supplementary material for: Cervical ripening in prolonged pregnancies by silicone double balloon catheter versus vaginal dinoprostone slow release system: The MAGPOP randomised controlled trial
Source: PLoS Med. 2021 Feb 11;18(2):e1003448. doi: 10.1371/journal.pmed.1003448 (PMC7877637; doi:10.1371/journal.pmed.1003448)
Supplement: S1 Data — (DOCX) [file pmed.1003448.s001.docx]

List of participating maternity units

- Pôle de gynécologie obstétrique, médecine fœtale, médecine et biologie de la reproduction, centre Olympe de Gouges, CHRU de Tours, Tours, France
- Maternité Centre hospitalier Regional d’Orléans, Orléans, France
- Department of Obstetrics and Gynecology, University Hospital of Nantes, Nantes, France
- Pôle de gynécologie obstétrique, hôpital Paule-de-Viguier, CHU de Toulouse, Toulouse, France
- Department of Obstetrics and Gynecology, Rene DUBOS Hospital, Cergy-Pontoise, France.
- Department of Gynecology and Obstetrics, University Hospital, Saint-Etienne, France
- CHU Brest, Hôpital Morvan, service de gynécologie-obstétrique, Brest, France
- Department of Obstetrics and Gynecology, Centre Hospitalier Departemental, la Roche sur Yon, France
- Pôle femme et enfant, CHU Estaing, Clermont-Ferrand
- Service de Gynécologie-Obstétrique, Reims, France
- Hôpital Saint Joseph, Department of Obstetrics and Gynecology, Marseille, France
- Service de gynécologie obstétrique et médecine de la reproduction, CHU de Caen, Caen, France
- Service de Gynécologie Obstétrique, Centre Hospitalier de Chartre, France
- Service de Gynécologie Obstétrique, Centre Hospitalier Universitaire de Rennes, France
- Department of Obstetrics and Gynecology, Poissy-Saint Germain Hospital, Poissy, France.
