# Supplementary figures and images for: Cervical ripening in prolonged pregnancies by silicone double balloon catheter versus vaginal dinoprostone slow release system: The MAGPOP randomised controlled trial
Source: PLoS Med. 2021 Feb 11;18(2):e1003448. doi: 10.1371/journal.pmed.1003448 (PMC7877637; doi:10.1371/journal.pmed.1003448)

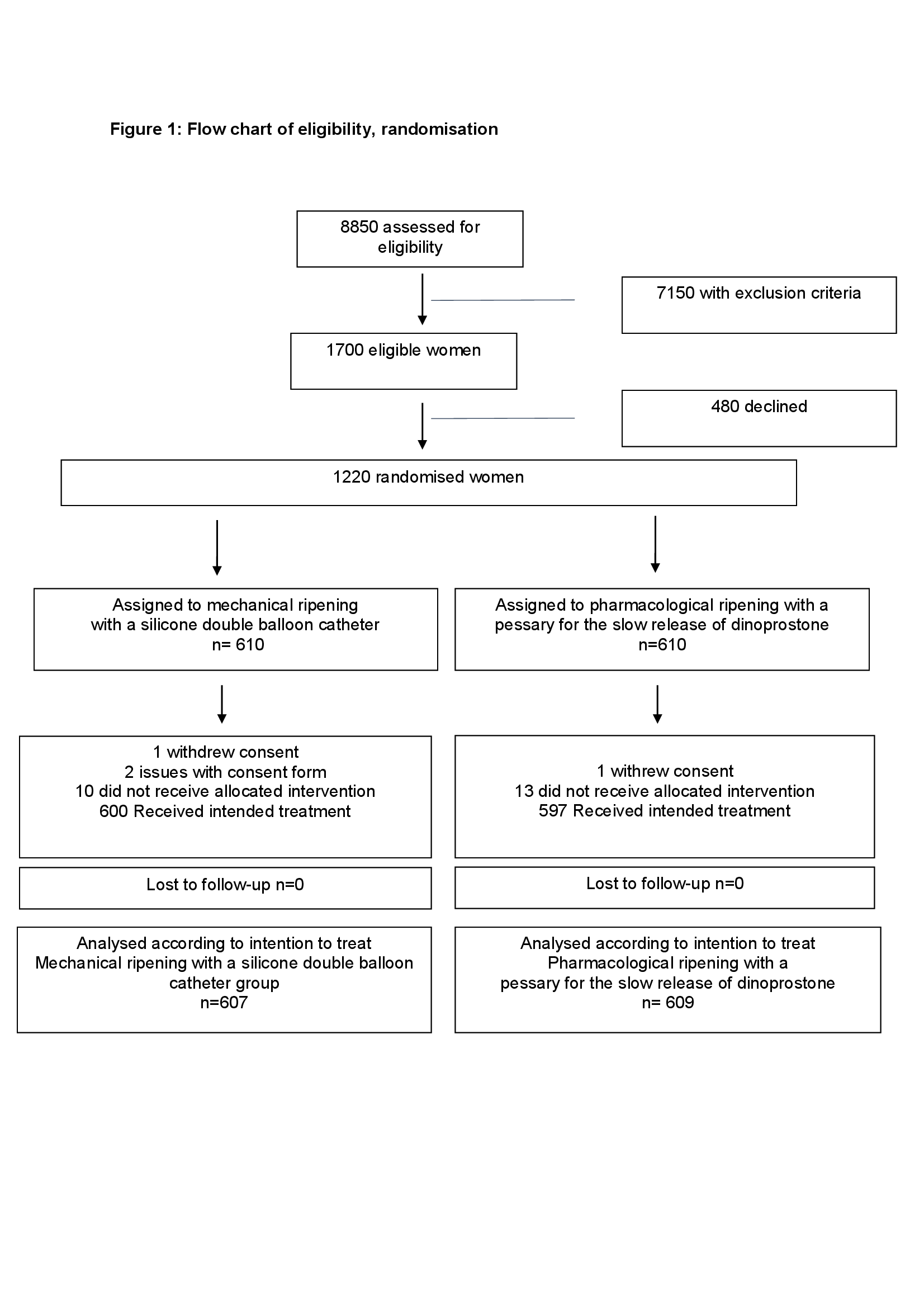

Supplement: S1 Fig — (TIFF) [file pmed.1003448.s002.tiff]
