# Supplementary material for: Cervical ripening in prolonged pregnancies by silicone double balloon catheter versus vaginal dinoprostone slow release system: The MAGPOP randomised controlled trial
Source: PLoS Med. 2021 Feb 11;18(2):e1003448. doi: 10.1371/journal.pmed.1003448 (PMC7877637; doi:10.1371/journal.pmed.1003448)
Supplement: S1 Table — (DOCX) [file pmed.1003448.s003.docx]

Table 1 Baseline characteristics of the intention to treat population, results are numbers and percentages unless otherwise stated

|  | **Mechanical group**  **(silicone double balloon catheter )**  n_1_=607 | **Pharmacological group**  **(pessary for the slow release of dinoprostone)**  n_2_=609 |
| --- | --- | --- |
| Age, years |  |  |
| Mean ± SD | 31.1 ± 5.2 | 31.3 ± 5.1 |
| Median (interquartile range) | 31.0 [27.4 ; 34.5] | 31.2 [27.5 ; 34.9] |
| Parity |  |  |
| Nulliparous | 401 (66.1) | 401 (65.8) |
| Ethnicity |  |  |
| *Sub-Saharan Africa* | 31 (5.1) | 45 (7.4) |
| *Asian* | 3 (0.5) | 1 (0.2) |
| *White* | 485 (80.0) | 471 (77.9) |
| *Hispanic* | 10 (1.7) | 12 (2.0) |
| *North African* | 77 (12.7) | 76 (12.6) |
| Pre-pregnancy weight, *kg* |  |  |
| Mean ± SD | 69.3 ± 16.2 | 68.3 ± 15.8 |
| Height, *cm* |  |  |
| Mean ± SD | 164.9 ± 6.4 | 165.3 ± 6.0 |
| Pre-pregnancy BMI, *kg/m^2^* |  |  |
| Mean ± SD | 25.4 ± 5.7 | 25.0 ± 5.4 |
| Pre-pregnancy obesity (BMI>30) | 109 (18.1) | 102 (16.8) |
| Last recorded weight, *kg* |  |  |
| Mean ± SD | 82.8 ± 15.7 | 82.1 ± 15.2 |
| Median (interquartile range) | 80.0 [72.0 ; 90.0] | 80.0 [72.0 ; 90.0] |
| Pre-existing diabetes (type 1 or 2) | 0 (0.0) | 0 (0.0) |
| Pre-existing Hypertension | 6 (1.0) | 2 (0.3) |
| Thyroid disease | 32 (5.3) | 38 (6.2) |
| Autoimmune disease | 5 (0.8) | 7 (1.1) |
| **Ongoing pregnancy** |  |  |
| Bishop score at inclusion |  |  |
| Mean ± SD | 3.2 ± 1.4 | 3.1 ± 1.4 |
| Median (interquartile range) | 3.0 [2.0 ; 4.0] | 3.0 [2.0 ; 4.0] |
| Smoking during pregnancy | 93 (15.3) | 95 (15.6) |
| Complication during pregnancy | 180 (29.7) | 180 (29.6) |
| Hypertension | 7 (1.1) | 7 (1.1) |
| Preeclampsia | 2 (0.3) | 0 (0.0) |
| Diabetes mellitus | 69 (11.4) | 88 (14.4) |
| Requiring insulin (percentages out of women with diabetes mellitus) | 12/69 (17.4) | 13/88 (14.8) |
| Threatened preterm labour | 4 (0.7) | 1 (0.2) |
| Intrahepatic cholestasis | 0 (0.0) | 0 (0.0) |
| Chronic disease decompensation | 3 (0.5) | 1 (0.2) |
| Small for gestational age | 26 (4.3) | 10 (1.6) |
| Indication for induction of labour (in addition to prolonged pregnancy) |  |  |
| Fetal heart rate abnormalities | 14 (2.3) | 12 (2.0) |
| Oligohydramnios | 99 (16.4) | 106 (17.5) |
| Reduced fetal movements | 36 (6.0) | 31 (5.1) |
| Hypertension | 14 (2.3) | 13 (2.2) |
| Preeclampsia maternal indication | 7 (1.2) | 2 (0.3) |
| Preeclampsia fetal indication | 1 (0.2) | 0 (0.0) |
| Fetus small for gestational age | 10 (1.7) | 5 (0.8) |
| Intrauterine growth restriction | 8 (1.3) | 4 (0.7) |
| Gestational diabetes | 28 (4.7) | 43 (7.1) |
| Abnormal Doppler findings | 2 (0.3) | 0 (0.0) |
| Gestational Thrombocytopenia | 3 (0.5) | 2 (0.3) |
| Maternal request | 10 (1.7) | 8 (1.3) |
| Other | 24 (4.0) | 28 (4.6) |
| SD standard deviation  BMI Body Mass Index | | |
