# Supplementary material for: Cervical ripening in prolonged pregnancies by silicone double balloon catheter versus vaginal dinoprostone slow release system: The MAGPOP randomised controlled trial
Source: PLoS Med. 2021 Feb 11;18(2):e1003448. doi: 10.1371/journal.pmed.1003448 (PMC7877637; doi:10.1371/journal.pmed.1003448)
Supplement: S2 Table — (DOCX) [file pmed.1003448.s004.docx]

Table 2 Outcomes related to cervical ripening and delivery results are numbers and percentages unless otherwise stated

|  | **Mechanical group**  **(silicone double balloon catheter )**  n_1_=607 | **Pharmacological group**  **(pessary for the slow release of dinoprostone)**  n_2_=609 | **Absolute difference* (95% CI)** | | ***p* **** |
| --- | --- | --- | --- | --- | --- |
| **Primary outcome** |  |  |  | |  |
| Caesarean section for non-reassuring fetal status | 35 (5.8) | 32 (5.3) | 0.5% [ -2.1; 3.1] | | 0.70 |
| **Secondary outcomes** |  |  |  | |  |
| Time from cervical ripening to delivery, hours |  |  |  | |  |
| Median (interquartile range) | 32.3 [23.3 ; 37.4] | 22.8 [14.8 ; 33.7] | 6.5 [5.0 ; 7.9] | | <0.001 |
| Delivery within the 12 hours after cervical ripening | 43 (7.4) | 98 (16.5) | -9.1 [-12.8 ; -5.5] | | <0.001 |
| Delivery within the 24 hours after cervical ripening | 157 (26.9) | 320 (53.9) | -26.9 [-32.3 ; -21.6] | | <0.001 |
| Need for induction with oxytocin | 503 (83.3) | 360 (59.2) | 24.1 [19.2 ; 29.0] | | <0.001 |
| Over whole oxytocin dose |  |  |  | | 0.85 |
| < 5 IU* | 365 (76.0) | 255 (74.3) |  | |  |
| ≥ 10 IU* | 15 (3.1) | 11 (3.2) |  | |  |
| More than 5 and less than 10 UI*** | 100 (20.8) | 77 (22.4) |  | |  |
| Uterine hyperstimulation | 7 (1.2) | 39 (6.4) | -5.2 [-7.4 ; -3.1] | | <0.001 |
| Tocolysis to treat hyperstimulation | 21 (3.5) | 39 (6.4) | -2.9 [-5.4 ; -0.5] | | 0.02 |
| Uterine Rupture | 0 (0) | 0 (0) |  | |  |
| Suspicious or pathologic fetal heart rate (FIGO 2015) | 241 (39.7) | 274 (45.0) | -5.3 [-10.8 ; 0.3] | | 0.06 |
| Analgesic use during labour | 165 (27.5) | 214 (35.4) | -7.9 [-13.2 ; -2.7] | | 0.003 |
| Antibiotics during labour | 126/597 (21.1) | 118/597 (19.8) | 1.3 [-3.2 ; 5.9] | | 0.57 |
| Caesarean delivery | 148 (24.5) | 143 (23.5) | 1.0 [-3.8 ; 5.8] | | 0.69 |
| Main indication for caesarean (% calculated out of the number of women who had a caesarean delivery) | | | | | |
| Non-reassuring FHR (defined by onsite physician) | 46 (31.1) | 56 (39.2) | |  |  |
| Cord prolapse | 3 (2.0) | 0 (0.0) | |  |  |
| Dystocic presentation | 5 (3.4) | 1 (0.7) | |  |  |
| Intrauterine infection | 4 (2.7) | 2 (1.4) | |  |  |
| Arrest of labour ≤ 5 cm | 49 (33.1) | 33 (23.1) | |  |  |
| Arrest of labour > 6 cm | 25 (16.9) | 36 (25.2) | |  |  |
| Non-engagement fetal head | 13 (8.8) | 11 (7.7) | |  |  |
| Failure of instrument-assisted delivery | 3 (2.0) | 4 (2.8) | |  |  |
| Vaginal delivery |  |  | |  |  |
| Operative vaginal delivery | 99/457 (21.7) | 130/466 (27.9) | | -6.2 [-11.8 ; -0.7] | 0.03 |
| Indication for operative vaginal delivery | | | | | |
| Failure to progress | 78/99 (78.8) | 93/130 (71.5) | |  |  |
| Fetal distress | 43 (43.4) | 74 (56.9) | |  |  |
| Cord prolapse | 0 (0.0) | 1 (0.8) | |  |  |
| Other | 1. 10.1) | 12 (9.2) | |  |  |
| Operative delivery | 247 (40.8) | 273 (44.8) | | -4.0 [-9.6 ; 1.6] | 0.16 |
| *Proportion difference for qualitative variables and median difference for quantitative variables  CI confidence interval  **Quantitative variables were compared with the Wilcoxon test, qualitative variables with the χ^2^ test  ***IU International Units | | | | | |
