# Supplementary material for: Cervical ripening in prolonged pregnancies by silicone double balloon catheter versus vaginal dinoprostone slow release system: The MAGPOP randomised controlled trial
Source: PLoS Med. 2021 Feb 11;18(2):e1003448. doi: 10.1371/journal.pmed.1003448 (PMC7877637; doi:10.1371/journal.pmed.1003448)
Supplement: S3 Table — (DOCX) [file pmed.1003448.s005.docx]

Table 3 Outcomes related to maternal morbidity

|  | **Mechanical group**  **(silicone double balloon catheter )**  N=605  n (%) | **Pharmacological group**  **(pessary for the slow release of dinoprostone)**  N=609  n (%) | **Proportion difference (95% CI)** | **p** |
| --- | --- | --- | --- | --- |
| Intrapartum infection* | 56 (9.4) | 49 (8.2) | 1.2 [-2.0 ; 4.4] | 0.47 |
| Postpartum infection* | 5 (0.8) | 2 (0.3) | 0.5 [-0.5 ; 1.6] | 0.29 |
| Postpartum haemorrhage** | 82 (13.6) | 95 (15.6) | -2.0 [-6.0 ; 1.9] | 0.31 |
| Transfusion ** | 7 (1.2) | 10 (1.6) | -0.5 [-1.8 ; 0.8] | 0.47 |
| 3rd or 4th degree tear** | 8 (1.3) | 9 (1.5) | -0.2 [-1.5 ; 1.2] | 0.82 |
| Admission to Intensive Care Unit* | 6 (0.9) | 3 (0.5) | 0.5 [-0.6 ; 1.7] | 0.34 |
| Fisher’s exact test*  χ^2^ test **  CI Confidence Interval | | | | |
