# Supplementary material for: Cervical ripening in prolonged pregnancies by silicone double balloon catheter versus vaginal dinoprostone slow release system: The MAGPOP randomised controlled trial
Source: PLoS Med. 2021 Feb 11;18(2):e1003448. doi: 10.1371/journal.pmed.1003448 (PMC7877637; doi:10.1371/journal.pmed.1003448)
Supplement: S4 Table — (DOCX) [file pmed.1003448.s006.docx]

Table 4 Outcomes related to neonatal morbidity results are numbers and percentages unless otherwise stated

|  | **Mechanical group**  **(silicone double balloon catheter )**  n_1_=605 | **Pharmacological group**  **(pessary for the slow release of dinoprostone)**  n_2_=609 | **Proportion difference (95% CI)** | ***p*** |
| --- | --- | --- | --- | --- |
| APGAR score at 5 min < 7 | 6 (1.0) | 11 (1.8) | -0.8 [-2.1 ; 0.5] | 0.23* |
| Umbilical arterial pH < 7 | 1 (0.2) | 5 (0.9) | -0.8 [-2.0 ; 0.3] | 0.22** |
| Hospitalisation of the neonate in intensive care | 41 (6.8) | 31 (5.1) | 1.7 [-1.0 ; 4.3] | 0.21* |
| Respiratory distress | 20 (3.3) | 12 (1.9) | 1.3 [-0.5 ; 3.1] | 0.15* |
| Neonatal asphyxia | 1 (0.2) | 2 (0.3) |  | - |
| *χ^2^ test  **Fisher’s exact test  CI Confidence Interval | | | | |
