# Supplementary material for: Cervical ripening in prolonged pregnancies by silicone double balloon catheter versus vaginal dinoprostone slow release system: The MAGPOP randomised controlled trial
Source: PLoS Med. 2021 Feb 11;18(2):e1003448. doi: 10.1371/journal.pmed.1003448 (PMC7877637; doi:10.1371/journal.pmed.1003448)
Supplement: Attachment — Submitted filename: response_to_reviewers.docx [file pmed.1003448.s008.docx]

Requests from the editors:

*Please structure your abstract using the PLOS Medicine headings (Background, Methods and Findings, Conclusions - "Methods and Findings" should be a single subsection). 

***The abstract was changed (page 2) as requested.***

*Ideally, in the last sentence of the Abstract Methods and Findings section, please include a brief note about any key limitation(s) of the study's methodology.

***This was done, the abstract conclusion was changed to (see end of page 2) “Observed caesarean rates for non-reassuring fetal status were much lower than those hypothesized during the trial planning. The difference in point estimates is 0.5 percentage points, in favour of the Propess group. The trial failed to demonstrate that mechanical ripening with a silicone double balloon catheter was superior to pharmacological cervical ripening with a pessary for the slow release of dinoprostone in reducing the rate of caesarean deliveries for non-reassuring fetal status in prolonged pregnancies.”***

*At this stage, we ask that you include a short, non-technical Author Summary of your research to make findings accessible to a wide audience that includes both scientists and non-scientists. The Author Summary should immediately follow the Abstract in your revised manuscript. This text is subject to editorial change and should be distinct from the scientific abstract. Please see our author guidelines for more information: <https://journals.plos.org/plosmedicine/s/revising-your-manuscript#loc-author-summary>

***This is the summary which was added (page 4) “When term is reached it may be necessary to induce labour. Induction of labour may require cervical ripening to increase the likelihood of a vaginal delivery. Two types of methods exist for cervical ripening but neither is specifically recommended for pregnancies that are prolonged. This trial compares mechanical methods with a silicone double-balloon catheter to pharmacological methods with a vaginal dinoprostone slow-release system among women with prolonged pregnancies. We did not find that mechanical methods were superior to pharmacological ones to reduce maternal and neonatal morbidity.”***

*The paper includes a CONSORT flowchart showing flow and inclusion of participants through the study but it would be good to also note that the reporting of the paper follows CONSORT guidelines (<http://www.consort-statement.org/>), and include a completed CONSORT checklist as supporting information alongside the revised paper when resubmitting. (When using the CONSORT checklist, of course update any relevant sections of the paper with more detailed methodological information as recommended by the reporting guideline).

***There is now a supplementary file called “consort checklist”.***

Reviewer #1:

The result is that there was no differecne between the methods. This is in accord with the latest systematic review.  The present trial is the largest reported but adds little to that review.

***Our population is a specific population of women with prolonged pregnancies, with a gestational age that was precisely estimated. The comparison was not made previously on such a population so we believe our result do add to this review.***

I note the conclusions of the Cochrane review. "Low- to moderate-quality evidence shows mechanical induction with a balloon is probably as effective as induction of labour with vaginal PGE2. However, a balloon seems to have a more favourable safety profile. More research on this comparison does not seem warranted. [...] Future research could be focused more on safety aspects for the neonate and maternal satisfaction."  

This trial does not so far as i can see report maternal satisfaction.

***We agree with the reviewer’s comment and regret that maternal satisfaction was not evaluated in our trial. We considered roughly interpreting maternal satisfaction from the secondary outcomes. Mechanical methods were associated with longer time between cervical ripening and delivery which has already proved to be associated with maternal dissatisfaction ( Dupont C et al. Dissatisfaction of women with induction of labour according to parity: Results of a population-based cohort study. Midwifery. 2020 Feb) which means mechanical methods could be associated with worst maternal satisfaction. But mechanical methods were also associated with reduced use of analgesic so could be better tolerated. Some pregnant women will prioritize analgesia, others will prioritize the speed of induction, so in the end maternal satisfaction was not evaluable through our secondary outcomes.***

***We are currently preparing a new trial on induction of labor in which we are planning to evaluate maternal satisfaction with questionnaires.***

However it does compare the balloon with the slow release dinoprosotne system (propess) which is arguably the safest form of prostaglandin.  It also adds to the safety profile of the balloon catheter which is valuable. 
The primary outcome is somewhat subjective, although an adjudication committee was used. As a safety check it would have been wise to report total caesarean rates to ensure that any differences were not just due to reclassifaction.  
I note that Total caesarean rate was not a secondary outcome.  However it is reported.  This is good.

***Indeed the total cesarean rates are reported in table 2. We observed no difference for the total cesarean rate between the two groups ( 24,5% in the mechanical group versus 23,5% in the pharmacological group; p=0,69). We have added this secondary outcome in the method section.***

Could the authors check that the secondary outocmes reported align with those prespecified on trial registry. If addtional outcomes are reported this should be noted.

***We have verified this and made some changes in the method section: “The caesarean delivery rate was added as a secondary outcome after the registration of the trial on clinicaltrials.gov.” (page 8)***

Remove the row multiparous in table 1 . it is redundant because the classes are mutually exclusive

***This was done (page 17)***

For prepregnancy weight, height and BMI remove the median IQR rows. Mean (SD) is better

***This was done (page 17)***

Remove mean (SD) apgar score in table 4.  Ditto mean and median UA pH

***This was done (page 22)***

Please remove duplicated results for text and tables. Remove them from the text.

***We have removed duplicated results, the result section is now much shorter (page 10)***

Please highlight the primary outocme in the relevant results table.

***The primary outcome is not in the tables, it is only in text. If the editors wish us to add it in the table we will.***

Reviewer #2:

The main limitation is that the primary event rate was only 1/3 of that anticipated, so it is underpowered to detect differences in this. 
- The conclusion that "Mechanical ripening was not superior to pharmacological cervical ripening in reducing the rate of caesarean deliveries for non-reassuring fetal status in prolonged pregnancies" cannot be made on this evidence, and should be tempered and discussed.

# *This difference between the anticipated rate and observed rate is frequent (Charles P et al Reporting of sample size calculation in randomized controlled trials: review, BMJ 2009) and does not necessarily mean the trial is underpowered, especially since 1200 women were included. We have tempered our discussion (page 11) to “The main limitation of the trial is that the observed rate of caesarean section for non-reassuring fetal status in the group treated pharmaceutically was much lower than expected. As a consequence, the a priori specified 5.7 percentage point difference (i.e., from 17.7 to 12%) appears debatable a posteriori. Nevertheless, the trial is large with 1216 women included in the analysis and point estimates are very close (5.8 vs 5.3%), and higher in the mechanical cervical ripening than in the pharmacological group”. Limitations of the trial due to this difference are already discussed in the following paragraph “Our a priori hypothesis was based on data from a trial conducted between 2009 and 2012 among women with prolonged pregnancies … recordings before the decision to perform a caesarean”*

***Our conclusion (page 12) was tempered to “Mechanical ripening with a silicone double balloon catheter was not found to be superior to pharmacological cervical ripening with a pessary for the slow release of dinoprostone in reducing the rate of caesarean deliveries for non-reassuring fetal status in prolonged pregnancies. This result does not provide evidence that justify preferring one strategy over the other.”***

Is there any way of including discussion of, given the extremely similar primary outcomes, how big a trial would have to be to detect differences/the likelihood of there being clinically meaningful differences?

***As the main outcome concerns only 5,3% of women in the control group, showing a reduction of the rate of the event would require to include a great amount of patients. We decided not to do this estimation as such a difference would not be clinically significant.***

A problem is interpretation the results. Based on MAGPOP, and the underpowered primary outcome.  I expect clinicians will use MAGPOP to support their preconceived views. Prostaglandin enthusiasts will quote the findings that labour is quicker, with less oxytocin and less failure to progress. Balloon catheter enthusiast will quote lower uterine hyperstimulation, less analgesia and clinician reported fetal distress. Discussion of results in the context of other RCTs may help -for example PROBAAT (Jozwiak M, Oude Rengerink K, Benthem M, et al. Foley catheter versus vaginal prostaglandin E2 gel for induction of labour at term (PROBAAT trial): an open-label, randomised controlled trial. Lancet. 2011;378(9809):2095-2103. doi:10.1016/S0140-6736(11)61484-0). Probaat had a very similar CS rate to MAGPOP trial, with both showing no difference in CS rate overall. Probaat also saw a statistically significant reduction in uterine hyperstimulation with mechanical methods. Could
meta-analysis of MAGPOP findings with other trial findings be considered as part of the paper? Or some kind of presentation of pros and cons in cases per 1000 (for every 1000 IOL there would be ..) to help women and clinicians make decisions about method of IOL.

***We believe a meta-analysis is a different subject and a subject in itself which cannot be treated in the same paper as the trial.***

***We were contacted by Dr M Jones and Pr Ben Mol who are planning an individual patient data meta anlaysis project to combine patient’s data from RCTs comparing vaginal prostaglandins to balloon catheters as methods for inducing labour (project on prospero). They hope to compare these two induction methods for specific delivery and safety outcomes. We have agreed to share our data and are very much looking forward to their results.***

Were any secondary outcomes adjudicated? For example, in view of clinician prior belief that PG are associated with hyperstimulation, there may have been a lower threshold to report and treat uterine hyperstimulation in the PG arm than the Ballon arm. If not adjudicated this should be discussed.

***Although a “cesarean for suspicious fetal heart rate” is a very subjective outcome and needed to be adjudicated other secondary outcomes were much more objective and did not require adjudication. For example “hyperstimuation” was defined in the protocol by a 6 contractions or more in 10 minutes over a 30 minutes period.***

***We have added in the methods section “secondary outcomes (which were not adjudicated)” to make the manuscript clearer (page 8).***

Also, it sounds like only CS were adjudicated. Is it possible that different proportions of vaginal births 'should' have been CS as indicated by objective review of fetal heart rate monitoring?

***We dreaded that some of the vaginal births should have been CS indicated for suspicious fetal heart rate. So we had planned that all the fetal heart rates of cases of neonatal asphyxia that occurred after a vaginal delivery would be reviewed by the adjudication committee to determine whether or not the fetal heart rate was suspicious enough to indicate an emergency cesarean section. In the end there were no cases of neonatal asphyxia after vaginal deliveries so this did not happen.***

The amount of missing data should be clearly stated, including the level of primary outcome data "imputed according to the worst case scenario method (assuming that women with missing data in the mechanical group had a caesarean for. non-reassuring FHR and that those in the pharmacological group did not). "

***This was added in the results section “In the end the main outcome was missing for two cases in the mechanical group” (page 10).***

As imputation of data was not stated in the protocol, this decision should be highlighted as being post hoc.

***Multiple imputation was specified in protocol but this method was not appropriate as missing data for the primary outcome were only in the mechanical group. We added this in the methods/ statistical analysis section “(as multiple imputation was not possible)”(page 9) and this in the results section “(which prevented us from doing multiple imputations)” (page 10).***

If levels of missing data were high a sensitivity analysis of compete case data could be considered.

***We have added the complete case analysis for the main outcome in the result section “A complete-case analysis observed no significant difference between the two groups (5.5% in the silicone double balloon catheter group compared vs 5.3% in the pessary for the slow release of dinoprostone group, difference in proportions 0.2%, 95% CI -2.3% to 2.7%, p=0.88)” (page 10).***


Generalisability to different settings should be discussed, in light of the protocols used.

***As explained in the discussion we believe our external validity for the French setting is strong “First, our inclusion of a large population from heterogeneous settings in different French regions in both university and general hospitals with public and private units that differ in size ensures strong external validity.”***

***However our results may not be generalisable to some settings (in particular in the Netherlands) where cervical ripening can be repeated during several days.***

It wasn't clear if clustering by centre was considered and how this was handled in analysis during analysis we searched for a center effect .

***Clustering by centre was analysed using a random logistic regression model and considered as a sensitivity analysis.***

Minor points: some clarifications would be useful e.g. "a gynaecologic position for its Insertion"  - not sure what this is.

***This was removed and made more simple “A speculum was inserted to facilitate cervical access, and the cervix wiped with a solution compatible with the woman’s allergies to prepare for device insertion”***

" cervix wiped with a solution compatible with the woman's allergies to prepare for device insertion"  - what are the options for solutions?

***We have added this precision in the methods section “povidone iodine or sodium hypochlorite” (page 6)***

Overall, I believe this manuscript should be published with i) more measured discussion of the limitations of the trial and ii) interpretation of findings in the context of other evidence. 
Data availability should be encouraged for transparency of the current analyses, and to allow meta-analysis with other studies to estimate effects with more precision - especially for some important secondary outcomes.

Reviewer #3:

Please confirm both interventions were used according to the manufacturer recommendations (or describe the deviations if they were not).

***We have added this to the manuscript “Both interventions were used according to the manufacturer’s recommendations.” (page 6)***

The study was a superiority trial but the trial did not show superiority. It would be worth a line or two about the impact of this on the conclusion that "there is no difference between the two treatments"

***The conclusion was changed to “Mechanical ripening with a silicone double balloon catheter was not found to be superior to pharmacological cervical ripening with a pessary for the slow release of dinoprostone in reducing the rate of caesarean deliveries for non-reassuring fetal status in prolonged pregnancies. This result does not provide evidence that justify preferring one strategy over the other.” (page 12)***

Please describe in more detail the justification for planning a primary outcome rate of 12% in the cervical balloon group

***We have changed the manuscript to “Accordingly we hypothesised that the caesarean rate for non-reassuring FHR would be 17.7% in the pharmacological group (pessary for the slow release of dinoprostone) and that mechanical cervical ripening (silicone double balloon catheter) would reduce the rate to 12%, which was considered to be a sufficient difference to justify recommending this method.” (page 9)***

It might also be worth mentioning that hyperstimulation higher in prostaglandin group.
***We have decided as suggested by the first reviewer to remove all duplicated results and we chose to leave the main outcome in the text and leave all secondary outcomes in the tables.***

Please comment on generalisability as mentioned above.

***As explained in the discussion and in the comments of the second reviewer we believe our external validity for the French setting is strong “First, our inclusion of a large population from heterogeneous settings in different French regions in both university and general hospitals with public and private units that differ in size ensures strong external validity.” However our results may not be generalisable to some settings (in particular in the Netherlands) where cervical ripening can be repeated during several days.***


Is the detailed protocol published on line somewhere - if so a link would be helpful.

***Yes, this is reference 16 of the manuscript Diguisto, C., Le Gouge, A., Giraudeau, B. & Perrotin, F. Mechanical cervicAl ripeninG for women with PrOlongedPregnancies (MAGPOP): protocol for a randomised controlled trial of a silicone double balloon catheter versus the Propess system for the slow release of dinoprostone for cervical ripening of prolonged pregnancies. BMJ Open 7, e016069 (2017).***

Reviewer #4:

One aspect I felt I didn't clearly understand was the generalisation from mechanical vs. pharmacological treatments. There is a hypothesis that from differences in uterine activity that mechanical treatments could be superior in avoiding caesarean deliveries. This seems reasonable to me (as a non-expert), but I couldn't follow why these two particular treatments were used, and whether they could be broadly seen as representative of other mechanical and pharmacological treatments that are available. It would be safer to specifically refer to these particular types of treatments rather than to the broad categories of  'pharmacological' vs. 'mechanical', and I am interested to see what the reviewers with expertise in this area think of this.

***We had initially planned to conduct a pragmatic trial to compare mechanical methods to pharmacological methods without choosing a specific agent or device for each method. All mechanical methods are equally efficient and have the same rates of side effects and at the time of the design of the trial all pharmacological methods were also equally efficient (since then oral misoprostol was authorised in France and it seems to be more efficient to achieve vaginal deliveries). So our initial aim was to compare mechanical and pharmacological methods without defining a single device or a single substance among the mechanical and pharmacological techniques. But for organisational reasons, because we had planned to have 15 participating units which could have used each different methods, this was not possible on a logistic perspective and too cumbersome on a regulatory perspective. So for organisational reasons and to standardise interventions, we chose the silicone double balloon catheter for the mechanical group and the Propess system for vaginal slow-release of dinoprostone for the pharmacological group.***

***We have changed the manuscript and do not refer to “mechanical methods” and “pharmacological methods” but* Mechanical ripening with a silicone double balloon catheter and Pharmacological ripening with a pessary for the slow release of dinoprostone (see throughout all manuscript)**

There were 17 secondary outcomes listed in the clinical trial registration. How was it decided which of these would be included in this main paper?

***All outcomes listed in the protocol are reported in the paper and some were added a posteriori as explained in the methods section as they seemed important to us “***… ***added as a secondary outcome after the registration of the trial on clinicaltrials.gov” (page 8)***

Was a detailed statistical analysis plan developed for this study before database lock/unblinding?

***Methods for data analysis were planned before unblinding the database. We have changed the manuscript to be more precise “A statistical analysis plan was finalized before the database be frozen”. (page 9)***

There are a very small number of (4 in total) reported as not analysed, what is the number (per treatment arm) who were analysed but didn't have the main or secondary outcomes? Could this information about missingness be included a supplementary table?

***We had one withdrawal of consent in each arm and 2 consent forms which were not complete and therefore who could not be included in the mechanical group. This is explained in the manuscript (page 10) “Due to two revocations of consent (n=2) and two issues with consent forms (n=2), 1216 women were analysed: 607 in the mechanical cervical ripening group (silicone double balloon catheter) and 609 in the pharmacological group (pessary for the slow release of dinoprostone). In the end the main outcome was missing for two cases in the mechanical group.”***

***For secondary outcomes we initially wrote the tables with the number of available data for each outcome but authors decided not to report the information as it was making the tables difficult to read and confusing. If the editor thinks the number of available data for every secondary outcome should be added in the tables, we will.***

The main analysis used a single-imputation strategy (caesarean for mech group, no caesarean for pharm group). This is reasonable approach, and if two more scenarios (oppose to worst-case scenario, and non-responder imputation (assume all missing patients had  caesareans) are included and show similar results this would demonstrate that the results are robust to any assumptions about missing data. 
***We had planned to do multiple imputation which was not feasible as the two missing data were in the mechanical group. As they were in the same group and in the mechanical one, the two methods (worst case scenario and non-responder imputation) showed the same results (imputation of a cesarean for the missing outcomes). In the end we chose to present the imputation with the non-responder strategy. We added this in the methods/ statistical analysis section “(as multiple imputation was not possible)”(page 9) and this in the results section “(which prevented us from doing multiple imputations)” (page 10).***

***We have added the complete case analysis for the main outcome in the result section (page 10)“An analysis was conducted on complete cases and no differences were observed between the two groups for the rates of caesarean for non-reassuring fetal status (5.5% in the mechanical group compared with 5.3% in the pharmacological group p=0.88).” The results are similar.***

What strategy was used for secondary outcomes with missing data?

***No imputation method was used for secondary outcomes.***

There are specific comments below but an overall comment overall was that I couldn't see which specific tests applied to which of the secondary outcome variables (e.g. in Table 2 footnotes could be included). I would also prefer to see an effect estimate (HR, OR, Mean difference) with a 95% CI presented for the study outcomes in the tables and throughout the abstract and main body. (proportion difference: 0.5% ; 95% confidence interval -2.1% to 3.1%, p=0.70).

***These absolute differences were added in the tables 2 to 4 (page 19-22).***

What was the proportion of patients recruited at each of the sites?

***The median number of patients recruited at each site was 81,5 [Q25,Q75 43-136]***

Randomisation.
What were the parity strata (no previous/previous)?

***Yes nulliparous versus others, this was added in the method section (page 6).***

Does 'varying sizes' mean random block size? What were the random block sizes used?

***Due to the open-label design, the block size was not fixed. The size varies from 14 to 2.***

Outcomes related to maternal morbidity.
Were these outcomes added before DB lock and unblinding?

***Yes, they were defined in the protocol***

Analysis
Should this be 'random intercept logistic regression'?

***Clustering by centre was analysed using a random logistic regression model and considered as a sensitivity analysis***

What was the criteria for using Chi-squared or Fisher? Observed or expected cell size?

***The choice between the two tests was made considering expected frequencies of at least 5 in each of the response categories.***

Fisher's exact test will be conservatively biased in a 2x2 table which doesn't have fixed margins, Barnard's test is a more powerful alternative when the margins are not fixed. There are also exact regression models that area also good alternatives where 
***Only 4 variables were analyzed with the Fischer’s exact test. This was added in footnote in the tables 2 to 4 (page 19-22). The analysis were conducted with Barnard’s test but found same results.***

What type of analysis was done for time to delivery?

***Time to delivery was analyzed using a Wilcoxon test.***

Were all deliveries completed within the 2 days of the time-frame detailed in the [clinicaltrials.gov](http://clinicaltrials.gov)? Within the following 48 hours of cervical ripening, est

Typically this would be done with a survival analysis where censoring could be accounted for.
Could the time to delivery be presented in Kaplan-Meir plots (in supplementary appendix if space is an issue)?

***This is the Kaplan-Meir plot for the variable “time between cervical ripening and delivery” which was conducted on 1177 women (583 in the mechanical group and 594 in the pharmacological group). This analysis was conducted only on 1177 women as some women did not receive the intervention (because labor started spontaneously) and did not have a t=0.***

***Follow up was ensured until the discharge of the new born (as stated in the protocol) so all women delivered and there was no censoring. For this reason conducting a survival analysis is identical to a Wilcoxon test.***

The sample size calculation assumed that caesarean section rate would be 17.7% and 12% in the two study arms, while the overall rate was closer to 5%. Was overall event-rate monitored during the study?

***There was no monitoring during the study and no intermediate analysis which could indeed have alerted us on this much lower rate of the outcome.***

While useful, randomisation stratified by centre is fairly typical for an RCT and I don't necessarily view this as a strength of study design.

***Practices vary widely between units in obstetrical care. We therefore consider that stratification by centre is a strength and left this sentence.***
